# Supplementary material for: Fabrication of arbitrary three-dimensional suspended hollow microstructures in transparent fused silica glass
Source: Nat Commun. 2019 Mar 29;10:1439. doi: 10.1038/s41467-019-09497-z (PMC6441035; doi:10.1038/s41467-019-09497-z)
Supplement: Supplementary file 1 — Supplementary Information [file 41467_2019_9497_MOESM1_ESM.pdf]

Supplementary Information

Fabrication of arbitrary three-dimensional suspended hollow microstructures in transparent fused silica glass

Kotz *et al.*

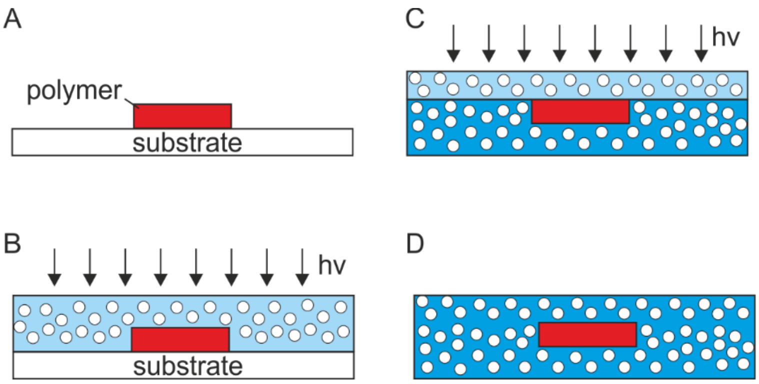

Supplementary Figure 1. Embodiment of microstructures inside the nanocomposite: A) Template structuring on a glass substrate. B) Casting and curing of the nanocomposite on the glass substrate. C) Closing the bottom of the structure by polymerizing another layer of the liquid nanocomposite onto the polymerized nanocomposite. D) Embedded polymeric structure.

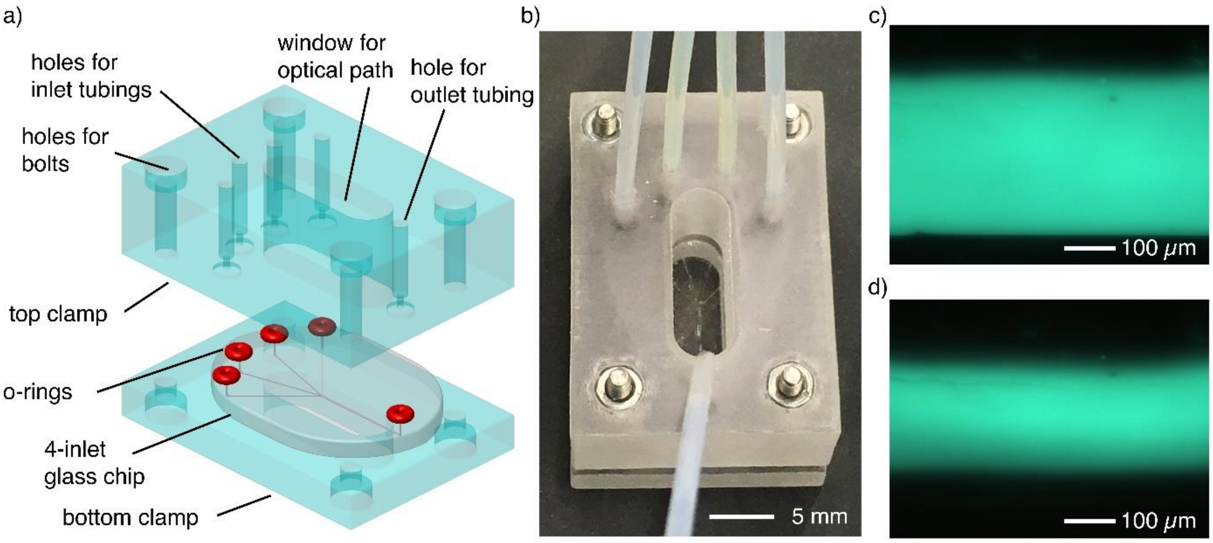

Supplementary Figure 2. **Hydrodynamic flow focussing**: A) Schematic illustration of 3D printed clamp, designed for connecting microfluidic tubing to glass chip. B) Picture of a 4-inlet and 1-outlet microfluidic glass chip after assembling between 3D printed clamp and connecting microfluidic tubings. (C) and (D) Fluorescence microscopy images showing hydrodynamic flow focusing of Fluorescein dye using 4-inlet and 1-outlet microfluidic glass chip where sheath flows (2 side inlets) are H_2O and middle streams (2 middle inlets) are aqueous solution of Fluorescein dye (10 µM). Flow rate ratio (FRR), calculated as total flow rate of sheath flows divided by total flow rate of middle streams is FRR=0 and FRR=1, respectively in (C) and (D).

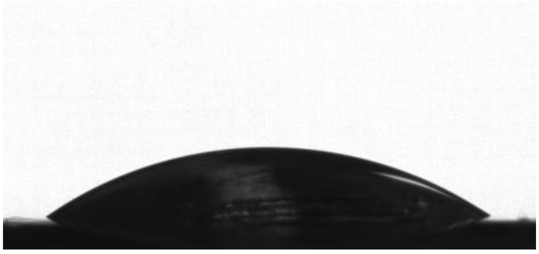

Supplementary Figure 3. Contact angle measurement: Sintered glass parts possess hydrophilic surface properties. 5 µl water droplet on a sintered glass surface showing a low contact angle of around 32 °.

Program	Temperature/°C	Heating rate/K/min	Holding phase/ min
Thermal debinding	150	0,5	120
	320	0,5	240
	600	0,5	120
	25	5	-
Sintering	800	3	90
	1300	3	120
	25	3	-

Supplementary Table 1. Thermal debinding and sintering protocol

The shrinkage during the heat treatment is isotropic and can be calculated in dependence of the solid loading using the following formula:

(1) $Y_s = 1 - (\Phi/(\rho_f/\rho_t))^{1/3}$

Φ – solid loading

ρ_f – final density

ρ_t – theoretical density”
